# FMRP regulates presynaptic localization of neuronal voltage gated calcium channels

**DOI:** 10.1016/j.nbd.2020.104779

**Published:** 2020-05

**Authors:** Laurent Ferron, Cesare G. Novazzi, Kjara S. Pilch, Cristian Moreno, Krishma Ramgoolam, Annette C. Dolphin

**Affiliations:** Department of Neuroscience, Physiology and Pharmacology, University College London, London WC1E 6BT, UK

**Keywords:** Calcium transients, Voltage-gated calcium channels, Trafficking, Synaptic transmission, Fragile X syndrome - FMRP

## Abstract

Fragile X syndrome (FXS), the most common form of inherited intellectual disability and autism, results from the loss of fragile X mental retardation protein (FMRP). We have recently identified a direct interaction of FMRP with voltage-gated Ca^2+^ channels that modulates neurotransmitter release. In the present study we used a combination of optophysiological tools to investigate the impact of FMRP on the targeting of voltage-gated Ca^2+^ channels to the active zones in neuronal presynaptic terminals. We monitored Ca^2+^ transients at synaptic boutons of dorsal root ganglion (DRG) neurons using the genetically-encoded Ca^2+^ indicator GCaMP6f tagged to synaptophysin. We show that knock-down of FMRP induces an increase of the amplitude of the Ca^2+^ transient in functionally-releasing presynaptic terminals, and that this effect is due to an increase of N-type Ca^2+^ channel contribution to the total Ca^2+^ transient. Dynamic regulation of Ca_V_2.2 channel trafficking is key to the function of these channels in neurons. Using a Ca_V_2.2 construct with an α-bungarotoxin binding site tag, we further investigate the impact of FMRP on the trafficking of Ca_V_2.2 channels. We show that forward trafficking of Ca_V_2.2 channels from the endoplasmic reticulum to the plasma membrane is reduced when co-expressed with FMRP. Altogether our data reveal a critical role of FMRP on localization of Ca_V_ channels to the presynaptic terminals and how its defect in a context of FXS can profoundly affect synaptic transmission.

## Highlights

• Loss of FMRP increases presynaptic Ca^2+^ transients.
• FMRP is a negative regulator of presynaptic Ca_v_2.2 channel abundance.
• FMRP reduces the forward trafficking of Ca_v_2.2 channels from ER to plasma membrane.
• Distal part of FMRP carboxy terminus is key for interaction with Ca_v_2.2 channels.

## Graphical abstract

**Figure:**
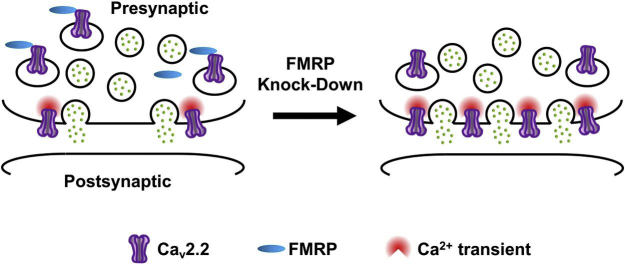

## 1 Introduction

Fragile X syndrome (FXS) is the most common form of intellectual disability and the leading known genetic cause of autism (Hagerman et al., 2017; Santoro et al., 2012). FXS is typically associated with cognitive, behavioral and social impairments as well as neurological abnormalities. Neuronal hyperexcitability is one of the typical features of neurological deficits in FXS (Contractor et al., 2015). FXS results from the transcriptional silencing of FMR1 gene and as consequence the loss of expression of the protein it codes for: the fragile X mental retardation protein (FMRP). FMRP is an RNA binding protein that controls the localization, stability and translation of numerous mRNAs critical to neuronal development, dendritic spine architecture and synaptic plasticity (For reviews see: Banerjee et al., 2018; Braat and Kooy, 2015; Contractor et al., 2015; Huber et al., 2015; Richter et al., 2015).

Recent studies have pointed out translational-independent functions for FMRP. Indeed, FMRP was shown to directly interact with ion channels and modulate neuronal excitability and neurotransmitter release (Brown et al., 2010; Deng et al., 2013; Ferron, 2016; Ferron et al., 2014; Yang et al., 2018). FMRP interacts with the sodium-activated potassium (Slack) channel and modulates its gating properties which regulates the excitability of bag cell neurons in *Aplysia* (Brown et al., 2010; Zhang et al., 2012). In CA3 hippocampal neurons, FMRP binds to beta-4 auxiliary subunits of Ca^2+^-activated potassium (BK) channels regulating its Ca^2+^ sensitivity and affecting the short-term plasticity at the CA3-CA1 synapse in mice (Deng et al., 2013; Deng et al., 2011). In cerebellar interneurons, FMRP interacts with K_V_1.2 channels to modulate GABA release (Yang et al., 2018). Finally, FMRP interacts with N-type voltage-gated Ca^2+^ channels modifying their cell surface expression and affecting their control of vesicular release in rat dorsal root ganglion (DRG) neurons (Ferron et al., 2014).

Ca^2+^ entry via voltage-gated calcium channels (VGCCs) triggers neurotransmitter release (For review see Neher and Sakaba, 2008). Multiple VGCC subtypes including P/Q- (Ca_V_2.1), N- (Ca_V_2.2) and R-type (Ca_V_2.3) mediate neurotransmitter release (Dolphin, 2012; Zamponi et al., 2015). Ca_V_2.1 channels play a major role in neurotransmission at mature synapses in the central nervous system whereas Ca_V_2.2 channels are predominant at synapses in the peripheral nervous system. Specific targeting of Ca_V_2 channels to subcellular compartments, including the active zone in presynaptic terminals, is critical for them to fulfil their function. In this study, we combined the use of two presynaptic functional markers (synaptophysin-GCaMP6f, sy-GCaMP6f, and vesicle-associated membrane protein - mOrange 2, VAMP-mOr2), one for Ca^2+^ transients and the second to indicate vesicular release, to investigate the impact of FMRP on the trafficking of Ca_V_ to the plasma membrane of active boutons. Here we show that the knock-down of FMRP increases the amplitude of the Ca^2+^ transient in functionally releasing presynaptic terminal of DRG neurons and that this effect is due to an increase of N-type Ca^2+^ channel contribution to the total Ca^2+^ transient. We also used live labelling techniques to show that FMRP controls cell surface expression of Ca_V_2.2 channels by regulating its forward trafficking between the endoplasmic reticulum (ER) and the plasma membrane. Altogether, our data show that FMRP is an important regulator of Ca_V_ trafficking and targeting to functional synapses and the loss of this regulatory mechanism likely contributes to neuronal hyperactivity observed in FXS.

## 2 Results

### 2.1 FMRP controls Ca^2+^ transients' amplitude in neuronal presynaptic terminals

We have previously shown that FMRP controls synaptic transmission via N-type Ca^2+^ channels in dorsal root ganglion (DRG) neuron terminals (Ferron et al., 2014) and we now wish to determine whether this effect is driven by a local accumulation of functional voltage-gated calcium channels.

**Fig. 1:**
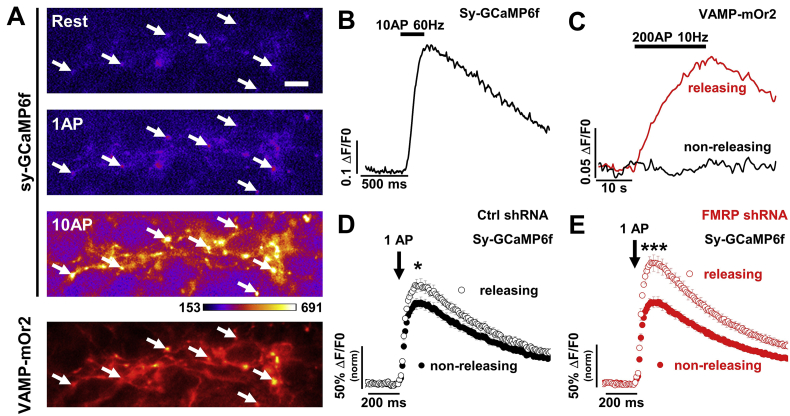
Effect of FMRP knock-down on Ca^2+^ transients in presynaptic terminals of DRG neurons. A) GCaMP6f fluorescence changes in presynaptic terminals of DRG neurons expressing sy-GCaMP6f and VAMP-mOr2, in response to electrical stimulation. White arrows point to some transfected boutons. Top three panels show sy-GCaMP6f fluorescence: at rest (top), after 1 AP (middle) and after 10 APs at 60 Hz (bottom). The pseudocolour scale is shown below the third panel. The bottom panel shows presynaptic terminals expressing VAMP-mOrange 2. Scale bar 5 μm. B) Example of increase of sy-GCaMP6f fluorescence (Ca^2+^ transients) in response to 10 APs at 60 Hz in DRG neuron terminals. The trace corresponds to the average response from 50 boutons. C) Example of variation of VAMP-mOr2 fluorescence in response to 200 APs at 10 Hz from DRG neuron terminals. Variations of VAMP-mOr2 fluorescence were used to identify vesicular release from presynaptic boutons: each individual bouton was analyzed and grouped into “non-releasing” (black trace, average of 15 boutons) or “releasing” (red trace, average of 35 boutons) groups depending on whether no variation or an increase of fluorescence was recorded in response to electrical stimulation. D) Sy-GCaMP6f fluorescence changes in response to 1 AP from non-releasing (black filled circles) and releasing (black open circles) presynaptic terminals of DRG neurons transfected with Ctrl shRNA. The Ca^2+^ transient was expressed as ΔF/F0 and normalized to the averaged peak recorded from non-releasing terminals (100.0 ± 7.2%, *n* = 31). The peak Ca^2+^ transient was increased to 122.8 ± 7.9% (n = 31, *P* = .045) in releasing terminals. Average sy-GCaMP6f responses (mean ± SEM) to 1 AP correspond to 5–6 trial averages from 25 to 50 boutons. n numbers correspond to independent experiments. * *P* < .05, one-way ANOVA and Bonferroni post-hoc test. E) Sy-GCaMP6f fluorescence changes in response to 1 AP from non-releasing (red filled circles) and releasing (red open circles) presynaptic terminals of DRG neurons transfected with FMRP shRNA. The Ca^2+^ transient was expressed as ΔF/F0 and normalized to the averaged peak recorded from non-releasing terminals (100.0 ± 6.8%, *n* = 34). The peak Ca^2+^ transient was increased to 149.6 ± 10.5% (*n* = 33, *P* = .00014) in releasing terminals. Average sy-GCaMP6f responses (mean ± SEM) to 1 AP correspond to 5–6 trial averages from 25 to 50 boutons. n numbers correspond to independent experiments. *** *P* < .001, one-way ANOVA and Bonferroni post-hoc test. (For interpretation of the references to color in this figure legend, the reader is referred to the web version of this article.)

To test this hypothesis, we monitored the local Ca^2+^ transient using the functional presynaptic reporter synaptophysin tagged with the genetically encoded Ca^2+^ indicator GCaMP6f: sy-GCaMP6f (Kadurin et al., 2016) (Fig. 1A). Sy-GCaMP6f positive nerve terminals were identified with a stimulus of 10 action potentials (APs) at 60 Hz (Fig. 1A and B). Rat DRG neurons co-cultured with dorsal horn (DH) neurons from embryonic stage 18 (E18) form functional synapses (Albuquerque et al., 2009; Ferron et al., 2014). In order to identify functionally releasing presynaptic terminals, E18 DRG neurons were co-transfected with a reporter of presynaptic exocytosis: VAMP tagged at its luminal carboxy terminal with the pH-sensitive fluorescent protein mOrange 2 (VAMP-mOr2; Fig. 1A). Increase of VAMP-mOr2 fluorescence in response to a stimulus of 200 APs at 10 Hz was used to identify releasing terminals (Fig. 1C). The impact of FMRP on local Ca^2+^ transients was then determined by knocking down its expression only in the presynaptic DRG neurons, by co-transfecting a short hairpin RNA (shRNA) (Ferron et al., 2014).

**Fig. 2:**
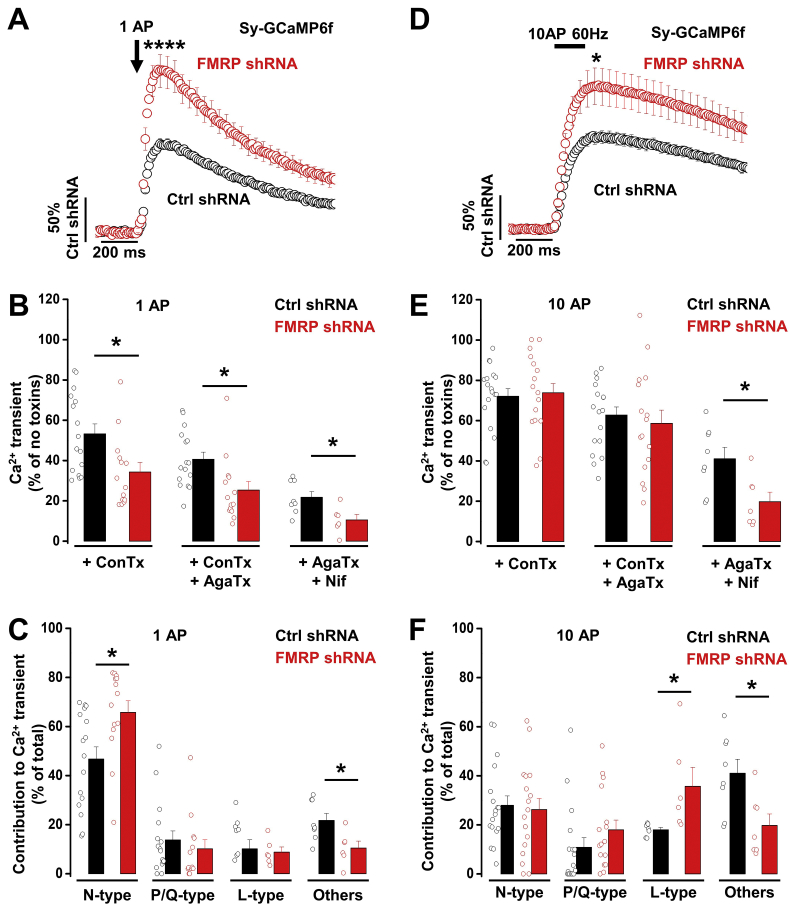
FMRP knock-down increases Ca^2+^ transients in presynaptic terminals of DRG neurons via N-type calcium channels. A) Average increase of sy-GCaMP6f fluorescence in response to 1 AP recorded from synaptic terminals transfected with either Ctrl shRNA (black circles) or FMRP shRNA (red circles). The Ca^2+^ transient was expressed as ΔF/F0 and normalized to the averaged peak in the Ctrl shRNA condition. Peak values are 100.0 ± 6.9% (n = 31) and 186.4 ± 17.9% (n = 33, *P* = .00004) for Ctrl shRNA and FMRP shRNA, respectively. Average sy-GCaMP6f responses (mean ± SEM) to 1 AP correspond to 5–6 trial averages from 25 to 50 boutons. n numbers correspond to independent experiments. ****P < .0001, one-way ANOVA and Bonferroni post-hoc test. B) Effect of sequential application of specific calcium channel blockers on the amplitude of the Ca^2+^ transient in response to 1 AP. Average Ca^2+^ transients were normalized to their respective “no toxin” peak in Ctrl shRNA and FMRP shRNA condition. The residual Ca^2+^ transient in response to 1 AP after treatment with ω-conotoxin GVIA (ConTx, 1 μM; N-type calcium channel blocker) was 53.3 ± 1.9% (*n* = 16) in Ctrl shRNA and 34.3 ± 4.8% (*n* = 14, *P* = .01) in FMRP shRNA. When ω-agatoxin IVA (AgaTx, 300 nM; P/Q-type calcium channel blocker) was added to the perfusion 40.7 ± 3.5% (n = 16) and 25.4 ± 4.3% (n = 14, *P* = .01) of the Ca^2+^ transient remained for Ctrl shRNA and FMRP shRNA, respectively. After adding nifedipine (Nif, 10 μM; L-type calcium channel blocker) to the perfusion, the remaining Ca^2+^ transient was 21.7 ± 2.9% (*n* = 8) and 10.5 ± 2.8% (*n* = 6, *P* = .016) for Ctrl shRNA and FMRP shRNA, respectively. Average sy-GCaMP6f responses (mean ± SEM) to 1 AP correspond to 5–6 trial averages from 25 to 50 boutons. n numbers correspond to independent experiments. * *P* < .05, one-way ANOVA and Bonferroni post-hoc test. C) Respective contribution of voltage-gated calcium channels to the Ca^2+^ transient in response to 1 AP in presynaptic terminals of DRG neurons. In the Ctrl shRNA condition, N-type, P/Q-type, L-type channels and other types contribute to 46.7 ± 4.9% (n = 16), 13.8 ± 3.7% (n = 16), 15.7 ± 2.8% (n = 8) and 21.7 ± 2.9% (n = 8), respectively. In FMRP shRNA condition, N-type, P/Q-type, L-type channels and other types contribute to 65.7 ± 4.8% (n = 14, *P* = .01), 10.1 ± 3.7% (*n* = 13, *P* = .49), 8.9 ± 2.1% (*n* = 6, *P* = .09) and 10.5 ± 2.8% (n = 6, *P* = .016), respectively. n numbers correspond to independent experiments. * *P* < .05, one-way ANOVA and Bonferroni post-hoc test. D) Average increase of sy-GCaMP6f fluorescence in response to 10 APs at 60 Hz, recorded from synaptic terminals transfected with either Ctrl shRNA (black circles) or FMRP shRNA (red circles). Ca^2+^ transient was expressed as ΔF/F0 and normalized to the averaged peak in the Ctrl shRNA condition. Peak values are 100.0 ± 7.0% (n = 31) and 150.0 ± 19.0% (n = 34, *P* = .02) for Ctrl shRNA and FMRP shRNA, respectively. Average sy-GCaMP6f responses (mean ± SEM) to 1 AP correspond to 5–6 trial averages from 25 to 50 boutons. n numbers correspond to independent experiments. *P < .02, one-way ANOVA and Bonferroni post-hoc test. E) Effect of specific calcium channel blocker application on the amplitude of the Ca^2+^ transient in response to 10 AP at 60 Hz. The remaining Ca^2+^ transient after treatment with ConTx GVIA was 72.1 ± 3.8% (*n* = 18) in Ctrl shRNA and 73.8 ± 4.6% (*n* = 17, *P* = .77) in FMRP shRNA. After application of AgaTx the remaining Ca^2+^ transient was 62.8 ± 4.0% (n = 18) and 58.7 ± 6.5% (n = 16, *P* = .59) for Ctrl shRNA and FMRP shRNA, respectively. After application of Nif, the remaining Ca^2+^ transient was 41.0 ± 5.7% (n = 8) and 19.8 ± 4.7% (*n* = 7, *P* = .013) for Ctrl shRNA and FMRP shRNA, respectively. n numbers correspond to independent experiments. * *P* < .05, one-way ANOVA and Bonferroni post-hoc test. F) Respective contribution of voltage-gated calcium channels to the Ca^2+^ transient in response to 10 AP at 60 Hz in presynaptic terminals of DRG neurons. In Ctrl shRNA condition, N-type, P/Q-type, L-type channels and other types contribute to 27.9 ± 3.8% (n = 18), 10.8 ± 3.9% (n = 17), 18.0 ± 1.0% (n = 7) and 41.0 ± 5.7% (n = 8), respectively. In FMRP shRNA condition, N-type, P/Q-type, L-type channels and other types contribute to 26.2 ± 4.6% (n = 17, *P* = .77), 17.9 ± 4.0% (n = 16, *P* = .21), 35.7 ± 7.7% (*n* = 6, *P* = .03) and 19.8 ± 4.7% (n = 6, *P* = .013), respectively. n numbers correspond to independent experiments. * *P* < .05, one-way ANOVA and Bonferroni post-hoc test. Open circles (black and red) represent individual experiments. (For interpretation of the references to color in this figure legend, the reader is referred to the web version of this article.)

We first focused on the Ca^2+^ transient generated by 1AP (Fig. 1D and E). In the control (Ctrl) shRNA condition, the amplitude of the Ca^2+^ transient in releasing boutons is ~20% larger (100.0 ± 7.2%, *n* = 31 vs 122.8 ± 7.9%, n = 31, *P* = .045) compared with non-releasing ones; interestingly this difference was increased to ~50% in the FMRP shRNA condition (100.0 ± 6.8%, *n* = 34 vs 149.6 ± 10.5%, *n* = 33, *P* = .00014). When we compared the amplitude of the Ca^2+^ transient from releasing boutons in the FMRP shRNA vs Ctrl shRNA condition (Fig. 2A), we found an increase of ~86% following knock-down of FMRP (from 100.0 ± 6.9% for Ctrl shRNA, n = 31, to 186.4 ± 17.9% for FMRP shRNA, n = 33, *P* < .0001).

In order to identify VGCC subtypes involved in the Ca^2+^ transient, we used specific blockers for the 3 main VGCC types involved in synaptic transmission in DRG neurons: N-type (ω-conotoxin GVIA, ConTx), P/Q-type (ω-agatoxin IVA, AgaTx) and L-type (nifedipine, Nif) (Fig. 2B). After ConTx application in the Ctrl shRNA condition, the remaining Ca^2+^ transient is 53% of the total amplitude indicating that N-type channels mediate 46.7% of Ca^2+^ entry (Fig. 2C). When AgaTx was added to the perfusion in addition to ConTx, the remaining Ca^2+^ transient represented 40.7% of the total transient which shows that P/Q type channels contribute 13.8% to the total Ca^2+^ entry (Fig. 2C). Finally, when Nif was added to the perfusion in addition to AgaTx (ConTx was omitted at this stage, however ConTx blockade is still effective as its effect on N-type current is irreversible (Boland et al., 1994; Takahashi and Momiyama, 1993; Wheeler et al., 1994)), the remaining Ca^2+^ transient was 21.7% of the total which shows that L-type contributes 15.7%, and other channels (R-type and T-type) contribute 21.7% of the total Ca^2+^ transient (Fig. 2C). The use of 10 μM of Nif is sufficient to produce a complete block of L-type channels (Fox et al., 1987a, Fox et al., 1987b; Regan et al., 1991); however, such a concentration of Nif may also produce a partial block of other calcium channels (Fox et al., 1987a; Perez-Reyes, 2003) which could slightly affect the relative contributions of L-type and other channels. In the FMRP shRNA condition (Fig. 2B and C), there was an increased contribution attributable to N-type channels of ~20% to ~66% (Fig. 2C), whereas the remaining Ca^2+^ transient after treatment with all the Ca^2+^ channel blockers was significantly reduced to ~11% (Fig. 2C). P/Q- and L-type contributions were not significantly reduced (Fig. 2C).

We then examined the effect of presynaptic FMRP knock-down on the Ca^2+^ transient generated by 10 APs (Fig. 2D). We found that the amplitude of total Ca^2+^ transients was also increased by ~50% in terminals lacking FMRP (Fig. 2D). However, the application of VGCC-specific blockers in the Ctrl shRNA condition indicated there was no differential effect of ConTx, and thus there was a reduced relative contribution of N-type channels compared with the 1AP response (Fig. 2E). Indeed, N-type channels only contributed to 28% of the total Ca^2+^ transient (~20% less than in the response to 1AP) (Fig. 2F). Conversely, the contribution of “other” channels was increased by 20%. In the FMRP shRNA condition, only the sensitivity to Nif was modified (Fig. 2E). The relative contribution of L-type channels was increased by 20%, whereas the contribution of “other” channels was reduced by 20% (Fig. 2F).

**Fig. 3:**
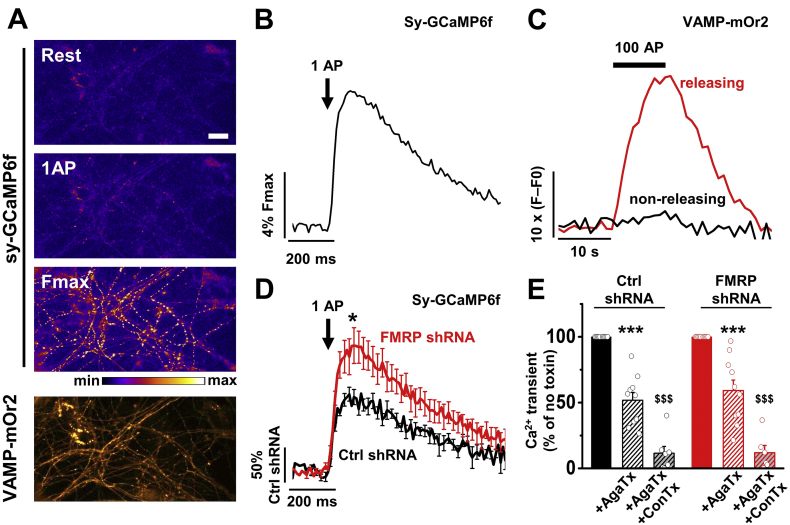
Effect of FMRP knock-down on Ca^2+^ transients from presynaptic terminals of hippocampal neurons. A) GCaMP6f fluorescence changes in presynaptic terminals of DRG neurons expressing VAMP-mOr2 and sy-GCaMP6f in response to electrical stimulation. Top three panels show sy-GCaMP6f fluorescence: at rest (top), after 1 AP (middle) and after ionomycin application (Fmax, bottom). Scale bar 20 μm. The pseudocolour scale is shown below the third panel. The bottom panel shows presynaptic terminals expressing VAMP-mO2. B) Example of increase of sy-GCaMP6f fluorescence (Ca^2+^ transients) in response to 1 AP from hippocampal neuron terminals. The trace corresponds to the mean response to 5 single APs from 50 individual boutons. The mean response was normalized to the maximum fluorescence (Fmax) obtained after application of ionomycin (5 μM). C) Example of variation of VAMP-mOr2 fluorescence (F - F0) in response to 100 AP at 10 Hz from hippocampal neuron terminals. Variations of VAMP-mOr2 fluorescence were used to identify vesicular release from presynaptic boutons: each individual bouton was analyzed and grouped into “non-releasing” and “releasing” categories when either no modification or an increase of fluorescence was recorded in response to electrical stimulation. D) Average increase of sy-GCaMP6f fluorescence in response to 1 AP recorded from pre-synaptic terminals transfected with either Ctrl shRNA (black trace) or FMRP shRNA (red trace). The Ca^2+^ transient was expressed as ΔF/F0 and normalized to the averaged peak in the Ctrl shRNA condition. Peak values are 100.0 ± 10.7% (*n* = 9) and 177.4 ± 25.5% (*n* = 10, *P* = .02) for Ctrl shRNA and FMRP shRNA, respectively. Average sy-GCaMP6f responses (mean ± SEM) to 1 AP correspond to 5–6 trial average from 50 to 75 boutons. n numbers correspond to independent experiments. *P < .05, one-way ANOVA and Bonferroni post-hoc test. E) Effect of specific calcium channel blocker application on the amplitude of the Ca^2+^ transient in response to 1 AP. Average Ca^2+^ transients were normalized to their respective no toxin peak in Ctrl shRNA and FMRP shRNA condition. The remaining Ca^2+^ transient in response to 1AP after treatment with AgaTx (300 nM; P/Q-type calcium channel blocker) was 51.8 ± 6.0% (n = 10) in Ctrl shRNA and 59.2 ± 8.0% (n = 10, *P* = .5, one-way ANOVA) in FMRP shRNA. In a subset of experiments, ConTx (1 μM; N-type calcium channel blocker) was added to the perfusion 11.5 ± 5.2% (n = 7) and 12.0 ± 5.5% (n = 6) of the Ca^2+^ transients remained for Ctrl shRNA and FMRP shRNA, respectively. Average sy-GCaMP6f responses (mean ± SEM) to 1 AP correspond to 5–6 trial average from 50 to 75 boutons. n numbers correspond to independent experiments. Open circles (black and red) represent individual experiments. *** *P* < .001, vs no toxin, paired *t*-test; ^$$$^*P* < .001, vs + AgaTx, one-way ANOVA and Bonferroni post-hoc test. (For interpretation of the references to color in this figure legend, the reader is referred to the web version of this article.)

We have previously shown that FMRP controls vesicular release in presynaptic terminals from hippocampal neurons (Ferron et al., 2014). We therefore also examined the effect of knock-down of FMRP on Ca^2+^ transients in response to 1 AP in terminals of hippocampal neurons in culture (Fig. 3A - 3D). Our data showed an increase in Ca^2+^ transients recorded from releasing presynaptic boutons in the FMRP knock-down condition by 77%, compared with the Ctrl shRNA condition (Fig. 3D), a similar result to that obtained in DRG-DH co-cultures.

We then used ConTx and AgaTx to determine the contribution of Ca_V_ channels to the Ca^2+^ transient. In the Ctrl shRNA condition, the AgaTx sensitive Ca^2+^ transient represented ~48% of the total Ca^2+^ transient and the addition of ConTx to the perfusion induced a further ~37% reduction (Fig. 3E). Our results indicated that only 11% of the other Ca_V_ channel types (L, R and T-type) contribute to the total Ca^2+^ transient. In the FMRP knock-down condition, the AgaTx sensitive Ca^2+^ transient represented ~41% of the total Ca^2+^ transient and the addition of ConTx induced a further ~47% reduction (Fig. 3E). Our results indicated that ~12% of the other Ca_V_ channel types contribute to the total Ca^2+^ transient. Overall, our results do not show a significant modification of the relative contribution of Ca_V_ channels to the total Ca^2+^ transient when FMRP is knocked-down, which suggests that the trafficking of both N- and P/Q-type channels is affected by FMRP in hippocampal neurons.

### 2.2 Distal FMRP C-terminal interacts with Ca_V_2.2 channels

**Fig. 4:**
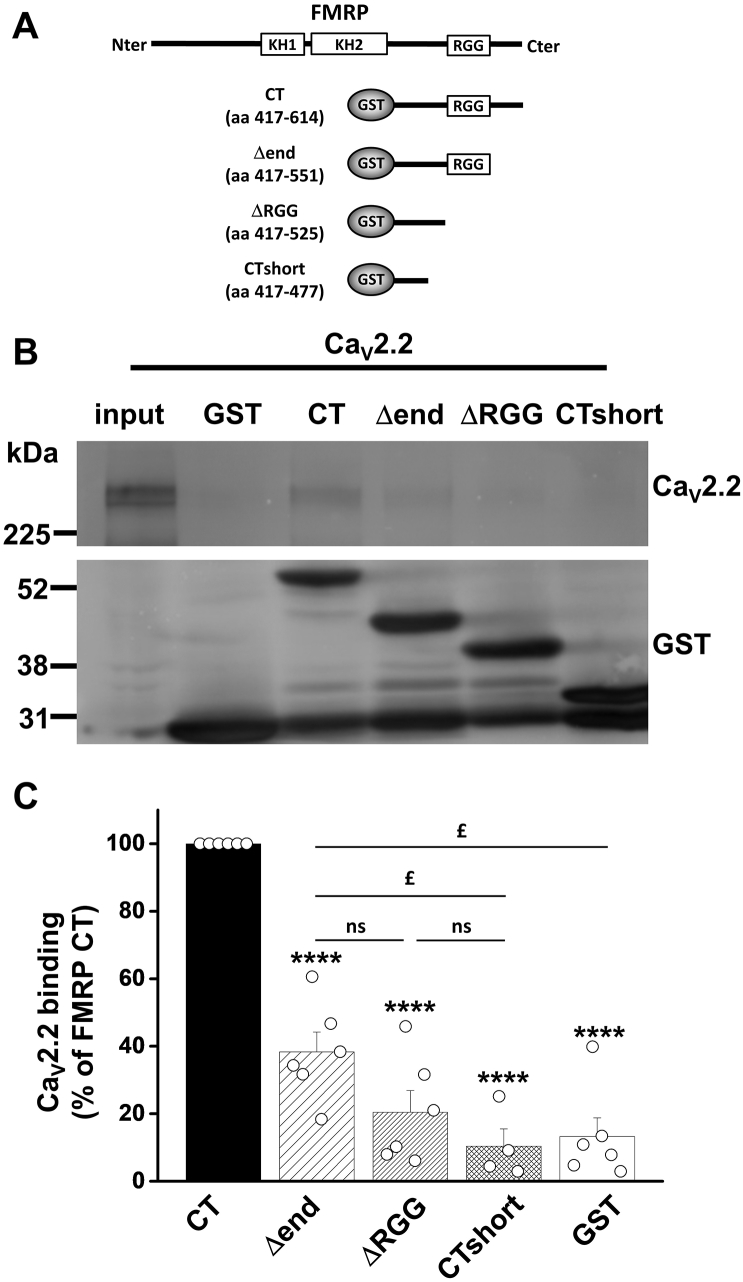
Distal FMRP C-terminus interacts with Ca_V_2.2. A) Schematic depiction of FMRP and GST-fusion fragments used for pull-down assay. Nter, N-terminus; Cter, C-terminus; KH1 and KH2, K-homology domains 1 and 2; RGG, arginine-glycine-glycine motif; aa, amino acid; CT, GST-FMRP C-terminus; Δend, GST-FMRP C-terminus deleted from the last 63 amino acids; ΔRGG, GST-FMRP C-terminus deleted from the last 89 amino acids which includes the RGG motif; CTshort, GST-FMRP C-terminus deleted from the last 137 amino acids. B) Western blots of pull-down assays show FMRP C-terminus binding Ca_V_2.2 expressed in tsA-201 cells compared with several deletant mutants for FMRP C-terminus and GST alone. Top panel shows immunoblots with Ca_V_2.2 II-III loop Ab. Lower panel shows immunoblots with GST Ab. Input represents 5% of protein input included in the assay. Representative of more than 4 independent experiments. C) Binding of Ca_V_2.2 expressed as a percentage of FMRP C-terminus (CT). Serial deletions of FMRP C-terminus resulted in 61.7 ± 5.8% (n = 6), 79.5 ± 6.4% (n = 6), 89.6 ± 5.1% (*n* = 4) and 86.7 ± 5.5% (n = 6) reductions of the binding for Δend, ΔRGG, CTshort and GST, respectively. n numbers correspond to independent experiments. Open black circles represent individual experiments. **** *P* < .0001 compared with CT, ^£^*P* < .05, ns: not significant, one-way ANOVA and Bonferroni post-hoc test.

We have previously identified a direct interaction between the C-terminus of FMRP and Ca_V_2.2 channels. Here, we aimed to identify the domain within the FMRP C-terminus involved in the FMRP/Ca_V_2.2 interaction. We generated glutathione S-transferase (GST) fusion proteins with serial deletions of the FMRP C-terminus (Fig. 4A). We applied whole-cell lysate from tsA-201 cells transfected with Ca_V_2.2/β1b/α_2_δ-1 to each purified GST-fusion protein and assessed their ability to interact with Ca_V_2.2 (Fig. 4B). We showed that the interaction between FMRP C-terminal and Ca_V_2.2 was strongly weakened by the deletion of the distal part of the FMRP C-terminus and then lost with the deletion of the RGG domain (Fig. 4B and C). Our data thus show that the distal domain of the FMRP C-terminus is crucial to the interaction with Ca_V_2.2.

### 2.3 FMRP reduces Ca_V_2.2 forward trafficking

**Fig. 5:**
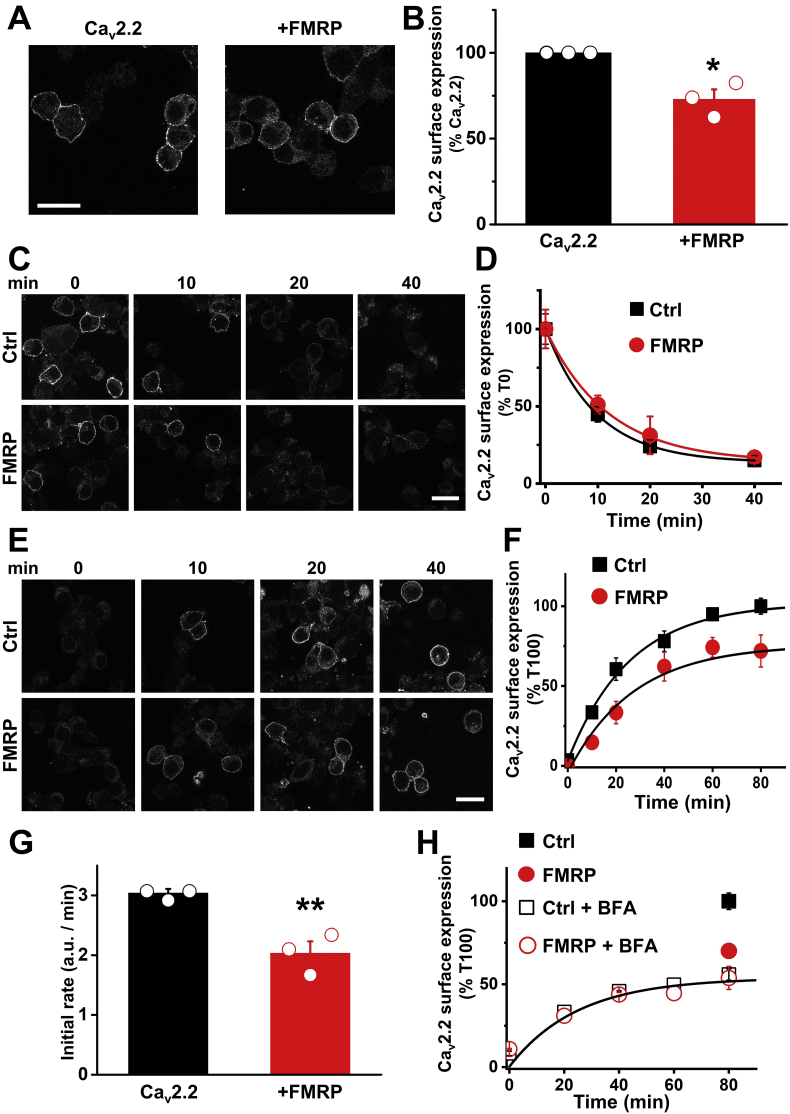
Endocytosis & forward trafficking. A) Representative confocal images of N2a cells expressing Ca_V_2.2-BBS 40 h after transfection and labelled with BTX-AF488. Cells were co-transfected with β1b, α2δ-1 and either empty vector (Ctrl, left panel) or FMRP (right panel). Cells were incubated at 17 °C with BTX-AF488 for 30 min and then fixed and imaged. Scale bar, 20 μm. B) Ca_V_2.2-BBS surface expression in Ctrl (black bar) and with co-expression of FMRP (red bar). BTX-AF488 fluorescence was reduced by 26% when FMRP was co-expressed (FMRP: 73.8 ± 5.8%, *n* = 3, *P* = .042, Paired *t*-test, n number corresponds to independent experiments). Solid bars are mean (± SEM) and open circles individual data points. C) Confocal images of N2a cells expressing Ca_V_2.2-BBS and labelled with BTX-AF488. Cells were co-transfected with β1b, α_2_δ-1 and either empty vector (Ctrl, top panels) or FMRP (bottom panels). Cells were incubated at 17 °C with BTX-AF488 for 30 min and then imaged at different time points, from 0 to 40 min after elevation to 37 °C. Scale bar, 20 μm. D) Time course of endocytosis of cell surface Ca_V_2.2-BBS in Ctrl (black squares) and + FMRP (red circles). BTX-AF488 fluorescence was normalized to the mean fluorescence at the time point 0 for each condition. The results are shown as the mean ± SEM (*n* > 120 cells per time point from 2 independent experiments). The data were fitted with single exponentials. The time constants of the fits were 9.7 ± 0.3 min and 12.0 ± 0.3 min for Ctrl and + FMRP, respectively. E) Confocal images of N2A cells expressing Ca_V_2.2-BBS and labelled with BTX-AF488. Cells were co-transfected with β1b, α2δ-1 and either empty vector (Ctrl, top panels) or FMRP (bottom panels). Cells were incubated at 17 °C with unlabelled BTX for 30 min, then incubated with BTX-AF488 at 37 °C and imaged at different time points, from zero to 80 min. Scale bar, 20 μm. F) Time course of insertion of Ca_V_2.2-BBS at the cell surface in Ctrl (black squares) and + FMRP (red circles). BTX-AF488 fluorescence was normalized to the mean fluorescence of the Ctrl condition at the time point 80 min. The results are shown as the mean ± SEM (n > 120 cells per time point from 3 independent experiments). The data were fitted with single exponentials. The time constants of the fits were 25.6 ± 2.4 min and 27.0 ± 5.3 min for Ctrl and + FMRP, respectively. G) Initial rates of net forward trafficking of Ca_V_2.2-BBS in Ctrl (black bar) and + FMRP (red bar). Rates of forward trafficking were determined by taking the slope of the linear phase between 0 and 20 min for each condition. Ctrl: 3.0 ± 0.1 a.u./min (n = 3 independent experiments) and FMRP: 2.0 ± 0.2 a.u./min (n = 3 independent experiments; ** *P* = .009, one-way ANOVA). Solid bars are mean (± SEM) and open circles individual data points. H) Time course of insertion of Ca_V_2.2-BBS into the cell surface in the presence of BFA in Ctrl (open black squares) and + FMRP (open red circles). Controls without BFA at 80 min are also shown (Ctrl, filled black square; +FMRP, filled red circle). The results are shown as mean ± SEM (*n* > 80 cells per time point from 2 independent experiments). The data were fitted with single exponentials. The time constants of the fits after treatment with BFA were 27.4 ± 2.7 min and 24.9 ± 4.4 min for Ctrl and + FMRP, respectively. The initial rates of net forward trafficking after treatment with BFA were 1.14 ± 0.01 a.u./min and 1.10 ± 0.03 a.u./min for Ctrl and + FMRP, respectively. (For interpretation of the references to color in this figure legend, the reader is referred to the web version of this article.)

We have shown that in DRG neurons lacking FMRP, the N-type VGCC-dependent Ca^2+^ transient was increased at presynaptic terminals. We have previously shown that cell surface expression of Ca_V_2.2 channels was reduced in tsA-201 cells over-expressing FMRP (Ferron et al., 2014). Cell surface expression of transmembrane proteins results from the balance between the trafficking of newly synthesized proteins from the endoplasmic reticulum to the plasma membrane (forward trafficking), internalization (endocytosis) from the plasma membrane to intracellular compartments and their recycling and/or degradation. In order to identify the mechanism of action of FMRP on Ca_V_2.2 cell surface expression, we have used Ca_V_2.2 channels with a tandem α-bungarotoxin binding site (BBS) tag in an extracellular loop (Ferron et al., 2014). We first checked that Ca_V_2.2-BBS cell surface expression was reduced when FMRP was co-expressed in Neuro2A (N2a) cells. After 2 days expression, N2a cells were live-labelled with fluorescently tagged α-bungarotoxin and the cell surface fluorescence was quantified (Fig. 5A). We found that Ca_V_2.2-BBS staining was reduced by 26% when FMRP was co-expressed (Fig. 5B). We then investigated the effect of FMRP on Ca_V_2.2 endocytosis by comparing the rate of internalization of Ca_V_2.2-BBS (Fig. 5C). Ca_V_2.2, with or without FMRP, showed similar kinetics of endocytosis (Fig. 5D). We next investigated the impact of FMRP on the net forward trafficking of Ca_V_2.2 by monitoring the insertion of new Ca_V_2.2-BBS into the plasma membrane over time (Fig. 5E). We found that the presence of FMRP reduced the initial speed of net forward trafficking of Ca_V_2.2 (extracted from the initial linear phase of the curve) from 3.0 ± 0.1 a.u. / min to 2.0 ± 0.20 a.u. / min (*n* = 3, *P* = .009) and led to a reduced steady-state maximum cell surface expression (Fig. 5F and G). Net forward trafficking results from the combination of newly synthesized proteins trafficked from the endoplasmic reticulum to the plasma membrane via the Golgi apparatus, and also from pre-existing proteins recycled from the plasma membrane and internal compartments. Brefeldin A (BFA) disrupts the structure of the Golgi apparatus and blocks the translocation of proteins from the endoplasmic reticulum to the plasma membrane. Forward trafficking experiments were repeated after treatment with BFA and we showed that the initial speed of forward trafficking of Ca_V_2.2 was reduced to 1.14 a.u./min and the steady-state maximum surface expression for Ca_V_2.2 was reduced to 50% in the Ctrl condition (Fig. 5H). Moreover, after BFA, Ca_V_2.2 forward trafficking characteristics were no longer different between the conditions with or without FMRP (Fig. 5H). These results indicate that FMRP has no impact on recycling of Ca_V_2.2 channels back to the plasma membrane, but instead acts on the forward trafficking of Ca_V_2.2 channels from the endoplasmic reticulum to the plasma membrane.

## 3 Discussion

Presynaptic Ca^2+^ influx plays a critical role in mediating neurotransmitter release (Dittman and Ryan, 2019). In this study we show that the knock-down of FMRP increases Ca^2+^ transients into presynaptic terminals of DRG neurons. Using specific calcium channel blockers, we demonstrate that this increase in Ca^2+^ transients is largely mediated by N-type Ca^2+^ channels. We also investigated the dynamic trafficking of Ca_V_2.2 channels and show that FMRP controls Ca_V_2.2 plasma membrane expression by reducing its forward trafficking between the ER and the plasma membrane. Altogether our data indicates that FMRP exerts a tight control on the functional expression of N-type Ca^2+^ channels at the synaptic nerve terminals.

We have previously shown that FMRP regulates vesicular release by modulating N-type Ca^2+^ channel density (Ferron et al., 2014). This regulation of vesicular release by FMRP could result from a modification of total Ca^2+^ influx (resulting from changes in VGCC gating and/or surface abundance) and/or changing in VGCC proximity to release sites (Dittman and Ryan, 2019). Thus, we investigated the effect of FMRP on the amplitude of Ca^2+^ transients and the VGCC subtype contribution in response to a brief stimulus (1AP, 1 ms) in DRG synapses onto DH neurons. Analysis of the response to 1AP revealed that N-type channels are, by far, the main contributors (~45%) to the total Ca^2+^ transient, and when FMRP was knocked-down their relative contribution increased further to 65%. Since we have previously shown that FMRP does not affect the biophysical properties of Ca_V_2.2 channels (Ferron et al., 2014), our data suggests that FMRP modulates vesicular release by controlling the abundance of Ca_V_2.2 channels at presynaptic terminals of DRG neurons. However, we cannot exclude that FMRP affects the proximity of VGCCs to the release sites and further experiments will be needed to shed light on this aspect.

We then carried out similar experiments on hippocampal neurons and revealed an equal contribution of N-type and P/Q-type channels to the presynaptic Ca^2+^ transient as previously demonstrated by Brockhaus & co-workers (Brockhaus et al., 2019). We also show that FMRP can control the trafficking of both N-type and P/Q-type channels to hippocampal synaptic terminals which is in good agreement with our previous study showing a direct interaction between FMRP and both Ca_V_2.1 and Ca_V_2.2 and an increase of vesicular release in hippocampal neurons when FMRP was knocked-down (Ferron et al., 2014).

It is interesting to note that the synaptic Ca_V_ contribution is distinct from that in the soma (Doughty et al., 1998) and it has been proposed that there is an independent regulation of the trafficking of Ca_V_2 channels to the active zone in presynaptic terminals (Cao et al., 2004; Cao and Tsien, 2010; Hoppa et al., 2012; Lubbert et al., 2019). The molecular mechanism controlling presynaptic Ca_V_2 channel accumulation and retention is still unknown and may depend on the type of synapse. It is tempting to suggest that FMRP might contribute to such a mechanism by controlling the targeting of Ca_V_2 channels to the active zone.

We also analyzed the impact of FMRP on Ca^2+^ transients generated by a sustained stimulus in DRG neuron terminals. Surprisingly, the response to 10 APs (60 Hz) revealed a very different pharmacological profile from the response to 1AP. Indeed, in control conditions, N-type channels only represented about 28% of the total Ca^2+^ transient, and the main source of Ca^2+^ (~40%) was triggered by R- and/or T-types Ca^2+^ channels (Bourinet et al., 2005; Wilson et al., 2000). This difference in contribution could be explained by several mechanisms that have been described to result from prolonged activity: 1) facilitation of R-type Ca^2+^ channels (David et al., 2010; Dietrich et al., 2003; Gomora et al., 2002; Leroy et al., 2003); and/or 2) secondary Ca^2+^ release from internal stores (de Juan-Sanz et al., 2017; Scott and Rusakov, 2006); and/or 3) deinactivation of T-type Ca^2+^ channels: at the resting membrane potential (between −55 and −65 mV for DRG neurons) (Du et al., 2014; Wang et al., 1994; Xu et al., 1997) most of the T-type Ca^2+^ channels are inactivated and only a small tail current can be generated by the remaining fraction of activatable channels during the repolarization phase of the AP. However, AP repolarization is followed by an after-hyperpolarization (AHP) lasting for several tens of milliseconds during which the membrane potential can reach values of −70 mV or below (Margas et al., 2016). During this hyperpolarization period, a larger fraction of T-type Ca^2+^ channels will become activatable and if a new AP is triggered during this AHP, which will occur for a 60 Hz stimulation (one AP every 17 ms), a much larger tail current will be generated by T-type Ca^2+^ channels. Altogether, these data suggest that facilitation of R-type Ca^2+^ channels, deinactivation of T-type Ca^2+^ channels and secondary Ca^2+^ release can contribute to the residual Ca^2+^ transient recorded in response to 10 APs. It is also worth mentioning that T-type Ca^2+^ channels are expressed in a small fraction of DRG neurons (Bernal Sierra et al., 2017; Watanabe et al., 2015) and that only a study using specific blockers will ascertain the involvement of these channels.

When FMRP was knocked down, the relative contribution of L-type channels to Ca^2+^ transients in response to 10 APs was increased by ~18% and the contribution of R-/T-type Ca^2+^ channels was proportionally reduced by ~21%. Interestingly, a study investigating the effect of the loss of FMRP on neuronal excitability in CA3 hippocampal neurons and in cortical pyramidal neurons has shown an excessive AP broadening and a reduction of the AHP amplitude during repetitive activity due to a reduced BK channel availability (Deng et al., 2013). Although such effects in DRG neurons lacking FMRP would still have to be demonstrated, we can speculate that during repetitive activity an extension of the repolarizing phase of the AP would allow a larger Ca^2+^ influx via L-type channels and a reduction of the AHP amplitude would limit the deinactivation of T-type channels and as a consequence reduce their contribution to the Ca^2+^ transient. Moreover, as mentioned above, the comparison of the pharmacological profile of the response to 1 and 10 APs indirectly showed that the increase of L-type channel contribution did not result from an increase in the number of channels at the plasma membrane but rather an increase in facilitation and/or secondary Ca^2+^ induced Ca^2+^ release. Interestingly, the mRNAs coding for CaMKII, which is involved in Ca^2+^-dependent facilitation for Ca_V_1.2 and Ca_V_2.1 channels (Hudmon et al., 2005; Jiang et al., 2008), and proteins involved in Ca^2+^ homeostasis (ryanodine receptor, IP3 receptor, sarcoendoplasmic reticulum Ca^2+^ ATPase) have all been identified as targets for FMRP (Darnell et al., 2011; Zalfa et al., 2003). Therefore, knock-down of FMRP could potentially change the expression of its target mRNAs in DRG neurons and account in part for the modification of Ca^2+^ elevation described here in presynaptic terminals. Supporting this idea, neuronal developmental defects have been linked to the dysregulation of intracellular Ca^2+^ dynamics (Ca^2+^ influx and release by the endoplasmic reticulum) in central nervous system neurons in a Drosophila model of FXS (Tessier and Broadie, 2011). Modulation of Ca^2+^ transients has often been reported in studies investigating the role of FMRP. However, the mechanism by which FMRP modulates Ca^2+^ transients appears distinct depending on the type of neuron and the developmental stage. Indeed, FMRP modulates Ca^2+^ transients by directly affecting VGCCs: upregulating L-type Ca^2+^ channels in dendritic spines of young (P14–23) mouse cortical neurons (Meredith et al., 2007) and downregulating them in the soma of neural progenitors derived from human induced pluripotent stem cells and mouse brain (Danesi et al., 2018); upregulating N-type Ca^2+^ channels and downregulating P/Q-type Ca^2+^ channels in the soma of mouse E14.5 primary cortical neurons in culture (Castagnola et al., 2018); and downregulating R-type Ca^2+^ channels in the soma of mouse E18 hippocampal neurons in culture (Gray et al., 2019). FMRP also modulates Ca^2+^ transients indirectly by affecting potassium channels: upregulating BK channels in the soma of CA3 hippocampal neurons in young mice (15–25 days), and in dendrites of somatosensory cortical pyramidal neurons in young mice (4–6 weeks) (Deng et al., 2013; Zhang et al., 2014), upregulating A-type K_V_4 channels in the dendrites of CA1 pyramidal neurons in adult mice (Routh et al., 2013); and upregulating K_V_1.2 channels in inhibitory interneurons in the cerebellum of young mice (26–32 days) (Yang et al., 2018).

FMRP interacts with Ca_V_2.2 channels via its C-terminal domain (Ferron et al., 2014). In the present study, we showed that the RGG domain (amino-acid 526 to 551) can interact with Ca_V_2.2 but we also showed that the critical domain involved in the interaction is the distal C-terminal part of FMRP (amino-acid 552 to 614). The C-terminal domain of FMRP harbors a Low Complexity Domain (LCD, residue 466–632 in human FMRP) including a short arginine-glycine-rich (RGG) region which is an important domain for the interaction with RNAs (for review see (D'Annessa et al., 2019)). LCDs are intrinsically disordered domains that can promote dynamic interactions with proteins and RNAs and have been implicated in the formation of ribonucleoprotein particles (Kato et al., 2012).

FMRP controls the expression and the activity of numerous ion channels either by regulating the translation of specific mRNAs (Darnell et al., 2011; Hagerman et al., 2017) or by interacting directly with the pore-forming subunit or one of their auxiliary subunits (Brown et al., 2010; Deng et al., 2013, 2019; Ferron et al., 2014; Yang et al., 2018). We have previously shown that FMRP affects the plasma membrane expression of Ca_V_2.2 (Ferron et al., 2014). In this study, we examined the effect of FMRP on the dynamic trafficking of Ca_V_2.2 channels to the plasma membrane. We have provided evidence that FMRP does not interfere with the endocytosis of Ca_V_2.2. Moreover, by disrupting the function of the Golgi apparatus with BFA, we have demonstrated that, while the recycling of the channels is not affected by FMRP, the forward trafficking of Ca_V_2.2 from the endoplasmic reticulum to plasma membrane is reduced by the co-expression of FMRP. Post-translational modifications of Ca^2+^ channels are important steps in controlling their trafficking to functional site (Dolphin, 2012; Huang and Zamponi, 2017; Lipscombe et al., 2013). We have previously shown that the reduction of Ca_V_2.2 cell surface expression induced by FMRP can be prevented by blocking proteasomal function, suggesting the involvement of the ubiquitin-proteasome system in the degradation of Ca_V_2.2 (Ferron et al., 2014). Ubiquitination is a common post-translational modification and it can influence synaptic efficiency by modifying the degradation, trafficking and the activity of ion channels (Abriel and Staub, 2005; Altier et al., 2011; Marangoudakis et al., 2012; Page et al., 2016; Waithe et al., 2011; Yi and Ehlers, 2007). The involvement of FMRP in modifying Ca_V_2.2 ubiquitination state will be investigated in future studies.

In summary, our findings reveal a critical role of FMRP in the localization of Ca_V_ channels to the presynaptic terminals and its effect on synaptic transmission in developing neurons. Controlling functional expression of Ca_V_ is currently under intensive study as it represents a potential therapy for many neurological diseases (Dolphin, 2018; Zamponi, 2016) and our findings suggest that it could also be a potential new avenue to restore proper synaptic plasticity and neural networks during early neural development in a context of FXS.

## 4 Methods

### 4.1 cDNA constructs

The following cDNAs were used: calcium channel Ca_V_2.2 (rabbit, GenBank: D14157), containing an extracellular HA tag or bungarotoxin binding site (Ferron et al., 2014), β1b (rat, GenBank: X61394), α_2_δ-1 (rat, GenBank: M86621). VAMP-mOrange2 was generated by replacing mCherry from pCAGGs-VAMP-mCherry by mOrange2 (gifts from Tim Ryan). Sy-GCaMP6f was made by replacing GCaMP3 in pCMV-SyGCaMP3 (a gift from Tim Ryan) by GCaMP6f (Chen et al., 2013). GFP-FMRP was provided by G. J. Bassell. Ctrl shRNA and FMRP shRNA were previously described (Ferron et al., 2008, 2014).

### 4.2 Cell culture and transfection

Mouse neuroblastoma N2A cells (ATCC, male sex) were cultured in Dulbecco's modified Eagle's medium (DMEM) and OPTI-MEM (1:1), supplemented with 5% fetal bovine serum (FBS), 1 unit/ml penicillin, 1 μg/ml streptomycin and 1% GlutaMAX (Thermo Fisher Scientific). tsA-201 cells (ECACC, female sex) were cultured in DMEM supplemented with 10% FBS, 1 unit/ml penicillin, 1 μg/ml streptomycin and 2% GlutaMAX (Thermo Fisher Scientific). Cell lines were cultured in a 5% CO_2_ incubator at 37 °C. tsA-201 cells were transfected using FuGENE 6 transfection reagent (Promega) according to the manufacturer's protocol. N2A cells were transfected using PolyJet (SignaGen) at a ratio of 3:1 to DNA mix according to manufacturer's instructions.

For primary neuron cultures, all experiments were performed in accordance with the Home Office Animals (Scientific procedures) Act 1986, UK, using a Schedule 1 method. DRG/DH co-cultures were prepared as previously described with minor modifications (Ferron et al., 2014). Decapitated embryonic Sprague Dawley rats (E18) were placed into ice-cold Leibovitz's L-15 medium. Spinal cords were removed and the dorsal thirds were placed in warm S-MEM containing trypsin (100 μl of 2.5% trypsin per ml of S-MEM) and incubated for 20–25 min at 37 °C. Digested tissues were then washed twice with warm growth medium (Neurobasal A, 2% B-27, 10% FBS, 1 unit/ml penicillin, 1 μg/ml streptomycin, 1% GlutaMAX and 100 ng/ml mouse nerve growth factor 7S (Thermo Fisher Scientific)) and gently triturated with fire-polished glass Pasteur pipette. The cell suspension was then plated onto poly-l-lysine/laminin treated glass coverslip and incubated at 37 °C in a 5% CO_2_ incubator. Dorsal root ganglia were also excised from E18 rats and placed in Hank's Basal Salt Solution (HBSS) containing 3.75 mg/ml dispase, 1000 U/ml DNase 1 (Thermo Fisher Scientific) and 0.8 mg/ml collagenase type 1A (Sigma) for 25–30 min at 37 °C in a shaking water bath (200 rpm). Digested tissues were washed with warm 10% FBS-HBSS and centrifuged at 500*g* for 5 min. The pellet was re-suspended in warm HBSS and triturated using fire-polished glass Pasteur pipette to produce a single cell suspension. The cell suspension was centrifuged at 500*g* for 5 min and resuspended in 100 μl of Nucleofector (Rat Neuron Nucleofector kit, Lonza) and electroporated with a cDNA mix (2 μg DNA containing: synaptophysin-GCaMP6f, VAMP-mOrange2 and either Ctrl shRNA or FMRP shRNA) according to the manufacturer's protocol. The electroporated cells were then incubated for 7 min in 10% FBS-RPMI containing 50 ng/ml NGF at 37 °C and finally re-suspended in growth medium to be added dropwise on top of the dorsal horn neurons. Two hours after plating, growth medium is added to the cells and 24 h later growth medium is replaced with 1.5 ml conditioned medium (50% growth medium and 50% conditioned rat cortical astrocyte medium). Forty-height 48 h after plating, uridine/5-fluoro-2′-deoxyuridine (5 μM) is added to the culture medium. Half of the culture medium is replaced every 4–5 days.

Hippocampal neurons were obtained from male P0 Sprague Dawley rat pups as previously described (Ferron et al., 2018; Meyer et al., 2019). Approximately 75 × 10^3^ cells in 200 μl of plating medium (MEM (Thermo Fisher Scientific) supplemented with B27 (Thermo Fisher Scientific, 2%), glucose (Sigma, 5 mg/ml), transferrin (Millipore, 100 μg/ml), insulin (Sigma, 24 μg/ml), fetal bovine serum (Thermo Fisher Scientific, 10%), GlutaMAX (Thermo Fisher Scientific,1%)) were seeded onto sterile poly-L-ornithine-coated glass coverslips. After 24 h, the plating medium was replaced with feeding medium (MEM supplemented with B27 (2%), glucose (5 mg/ml), transferrin (100 μg/ml), insulin (24 μg/ml), fetal bovine serum (5%), GlutaMAX (1%) and cytosine arabinose (Sigma, 0.4 μM)) half of which was replaced every 7 days. At 7 days in vitro (DIV) and 2 h before transfection, half of the medium was removed, and kept as ‘conditioned’ medium, and fresh medium was added. The hippocampal cell cultures were then transfected with synaptophysin-GCaMP6f, VAMP-mOrange2 and either Ctrl shRNA or FMRP shRNA using Lipofectamine 2000 (Thermo Fisher scientific). After 2 h, the transfection mixes were replaced with feeding medium consisting of 50% ‘conditioned’ and 50% fresh medium.

### 4.3 GST pull down assay

For pull-down assays, glutathione S-transferase (GST) was subcloned into pYES2.1/V5-His TOPO® TA (Invitrogen) by inserting PCR product using pGEX-2T as a template (GE Healthcare). GST-tagged constructs were generated by inserting PCR products of the mouse FMRP C-terminal (nucleotides 1514–2104; primer F: ACTAGT**GAATTC**TATATCACCTGAACTATTTAAAGGAAGTAGACC; primer R: ACTAGT**GAATTC**TTAGGGTACTCCATTCACCAGCGG), Δend (nucleotides 1514–1915; primer R: ACTAGT**GAATTC**TTATCCTTTGAAGCCTCCTCCTCT), ΔRGG (nucleotides 1514–1837; primer R: ACTAGT**GAATTC**TTACAGGAAGCTCTCCCTCTCTTC) and CTshort (nucleotides 1514–1693; primer R: ACTAGT**GAATTC**TTAATTTCTGTAAGGTCTACTACC) into *Eco*RI site of a pYES2.1/V5-His-GST. Yeasts (*Saccharomyces cerevisiae*) were transformed with individual expression vectors encoding the GST-fusion proteins and produced by standard methods. The yeast was lysed by vigorous shaking in PBS containing protease inhibitors (cOmplete tablet, Roche) and glass beads (Sigma) at 4 °C for 20 min. The lysates were then clarified by centrifugation (14,000 ×*g*, 5 min, 4 °C). GST-fusion proteins were immobilized on glutathione sepharose 4B beads (GE Healthcare) and incubated at 4 °C with lysate from tsA-201 cells transfected with Ca_V_2.2/α_2_δ-1/β1b. Beads were washed four times with ice-cold 1% Triton-PBS containing protease inhibitors (cOmplete tablet, Roche) and incubated for 15 min at 55 °C with 100 mM dithiothreitol and 2xLaemmli sample buffer. Eluted proteins were then resolved by SDS-PAGE. The following antibodies (Ab) were used: rabbit polyclonal anti- Ca_V_2.2 (Raghib et al., 2001) and mouse monoclonal anti-GST (Santa Cruz Biotechnology).

### 4.4 Western blot analysis

Forty-height hours after transfection, cells were rinsed twice with PBS and then harvested in PBS containing protease inhibitors (cOmplete tablet, Roche). The cells were lysed in PBS containing 1% Igepal and protease inhibitors for 30 min on ice. The detergent lysates were then clarified by centrifugation (14,000 ×*g*, 30 min, 4 °C). Proteins were separated by SDS-PAGE on 3–8% Tris-Acetate or 4–12% Bis-Tris gels and then transferred to polyvinylidene fluoride membranes. After blocking in TBS buffer (10 mM Tris, pH 7.4, 500 mM NaCl. 0.5% Igepal, 10% goat serum and 3% BSA), the membranes were incubated with primary antibody overnight. The protein-Ab complexes were then labelled with a horseradish peroxidase-conjugated secondary Ab (Sigma-Aldrich) for 1 h at room temperature and detected using the enhanced ECL Plus reagent (GE Healthcare) visualized with a Typhoon 9410 scanner (GE Healthcare). Quantification of immunoblot bands was performed with ImageQuant software (GE Healthcare) or Image J.

### 4.5 Endocytosis and forward trafficking experiments

N2a cells were plated onto glass-bottomed dishes (MatTek Corp., Ashland, MA) precoated with poly-l-lysine and transfected with a Ca_V_2.2 construct tagged with a double bungarotoxin binding site epitope (Ca_V_2.2-BBS) (Cassidy et al., 2014; Dahimene et al., 2018), α2δ-1, β1b and either empty vector or HA-FMRP (Ferron et al., 2014). After 40 h expression, cells were washed twice with Krebs-Ringer solution with HEPES (KRH) (in mM; 125 NaCl, 5 KCl, 1.1 MgCl_2_, 1.2 KH_2_PO_4_, 2 CaCl_2_, 6 Glucose, 25 HEPES, 1 NaHCO_3_). For endocytosis experiments, cells were incubated with 10 μg/ml α-bungarotoxin Alexa Fluor® 488 conjugate (BTX488) (Thermo Fisher Scientific) at 17 °C for 30 min. The unbound BTX488 was removed by washing with KRH, and the labelled cells were returned to 37 °C for the kinetic assay. Endocytosis was terminated by fixing the cells with cold 4% PFA-sucrose in PBS at the specified time. The cells were then mounted with VectaShield mounting medium (Vector Laboratories). For forward trafficking assay, the cells were incubated with 10 μg/ml unlabeled α-bungarotoxin (BTX; Invitrogen) at 17 °C for 30 min. The unbound BTX was washed off with KRH, and the cells were then incubated with 10 μg/ml BTX488 in KRH at 37 °C. To stop the reaction, cells were washed twice with cold KRH and then fixed with 4% PFA in PBS at specified times for the kinetic assay. Brefeldin A [BFA; 200 ng/ml (0.71 μM); Sigma-Aldrich] in 0.4% DMSO was added to the cells in FBS-free N2a cell culture medium for 4 h before the experiment, and during the experiment in KRH buffer. N2A cell samples were viewed on an LSM 780 confocal microscope (Zeiss) using a 63×/1.4 numerical aperture oil-immersion objective in 16-bit mode. The tile function (3 × 3 tiles, each tile consisting of 1024 × 1024 pixels) was used and every transfected cell within the image was analyzed to remove collection bias. Acquisition settings, chosen to ensure that images were not saturated, were kept constant for each experiment.

### 4.6 Live cell imaging

Neurons were imaged 14–16 days in culture. Live cell images were acquired as previously described with minor modifications (Kadurin et al., 2016). Coverslips were mounted in a rapid-switching, laminar-flow perfusion and stimulation chamber (RC-21BRFS, Warner Instruments) on the stage of an epifluorescence microscope (Axiovert 200 M, Zeiss). Live cell images were acquired with an Andor iXon+ (model DU-897U-CS0-BV) back-illuminated EMCCD camera using OptoMorph software (Cairn Research, UK). White and 470 nm LEDs served as light sources (Cairn Research, UK). Fluorescence excitation and collection was done through a Zeiss 40 × 1.3 NA Fluar objective using 450/50 nm excitation and 510/50 nm emission and 480 nm dichroic filters (for sy-GCaMP6f) and a 545/25 nm excitation and 605/70 nm emission and 565 nm dichroic filters (for mOrange2). Action potentials were evoked by passing 1 ms current pulses via platinum electrodes. Cells were perfused (0.5 ml min^−1^) in a saline solution at 25 °C containing (in mM) 119 NaCl, 2.5 KCl, 2 CaCl_2_, 2 MgCl_2_, 25 HEPES (buffered to pH 7.4), 30 glucose, 10 μM 6-cyano-7-nitroquinoxaline-2,3-dione (CNQX) and 50 μM D,L-2-amino-5-phosphonovaleric acid (AP5, Sigma). Images were acquired at 100 Hz over a 512 × 266 pixel area in frame transfer mode (exposure time 7 ms) and analyzed in ImageJ (http://rsb.info.nih.gov/ij) using a custom-written plugin (http://rsb.info.nih.gov/ij/plugins/time-series.html). Successfully transfected neurons were identified by visualizing sy-GCaMP6f fluorescence in response to a 33 Hz stimulation for 180 ms every 4 s. Subsequently, single stimulations of 1 ms (mimicking single AP) were repeated 5 times with 30–45 s intervals. Regions of interest (ROI, 2 μm diameter circles) were placed around synaptic boutons responding to an electrical stimulation of 10 AP at 60 Hz. Functional synaptic boutons were identified by the increase of fluorescence of VAMP-mOr2 in response to 200 APs at 10 Hz (in this case images were acquired at 2 Hz with 50 ms exposure time). ω-conotoxin GVIA (1 μM), ω-agatoxin IVA (300 nM) (Alomone Labs) and nifedipine (10 μM, Sigma, dissolved in DMSO) were perfused for at least 5 min either alone or in combination before imaging. In order to show that the expression of the Ctrl shRNA construct did not affect Ca_V_ activity, we have compared the amplitude of the response to 1 AP (ΔF/F0) recorded from hippocampal neurons expressing sy-GCaMP6f and VAMP-mOr2 vs hippocampal neurons expressing sy-GCaMP6f and VAMP-mOr2 with Ctrl shRNA: 0.030 ± 0.005 (*n* = 5) and 0.034 ± 0.004 (n = 5), respectively (*P* = .57, *t*-test).

### 4.7 Statistical analysis

Data are given as mean ± SEM. Statistical comparisons were performed using paired, unpaired Student's *t*-test or one-way ANOVA with Bonferroni post-hoc test, as appropriate, using OriginPro 2016.

## Declaration of Competing Interest

None.
